# Usage of Natural Health Products (NHPs) for respiratory diseases: user characteristics and NHP-Consumption behavior during the Covid-19 pandemic in Germany

**DOI:** 10.1186/s12906-023-04180-9

**Published:** 2023-10-21

**Authors:** Miriam Wolf, Agnes Emberger-Klein, Klaus Menrad

**Affiliations:** Department of Marketing and Management of Biogenic Resources, Campus Straubing for Biotechnology and Sustainability, Hochschule Weihenstephan-Triesdorf (University of Applied Sciences), Technical University of Munich, Am Essigberg 3, D-94315 Straubing, Bavaria, Germany

**Keywords:** Natural health product, Respiratory diseases, Self-medication, Consumer characteristics, Consumer behavior

## Abstract

**Background:**

Respiratory diseases (RD) can challenge healthcare systems around the globe. Natural health products (NHPs) are popular complementary and alternative medicine options for health issues concerning non-fatal RD. Little is known about the characteristics of the users of RD-NHPs and about their NHP consumption behavior during the Covid-19 pandemic in Germany.

**Methods:**

A representative online survey was conducted in Germany in 2022. 1707 participants were classified based on having used NHPs for RD within the previous 12 months, having used NHPs but not for RD within the previous 12 months and not having used NHPs. Data were analyzed using descriptive and inferential statistical methods as well as a multinomial logistic regression model.

**Results:**

Users of RD-NHPs within the previous 12 months were more likely to be employed and to consult pharmacists more often for non-fatal health issues than individuals who did not take RD-NHPs. RD-NHP users were more likely to suffer from a Covid-19 infection and to have children living in the same household than other NHP users. Compared to non-NHP users, RD-NHP users were more likely to be female, highly educated and have stronger openness-to-change value orientations. Vaccination-related behavior was no indicator of RD-NHP usage. Most RD-NHP users took NHPs in self-medication. Few reported informing their practitioner about their self-medication. Drugstores were the most visited supply source for NHPs during the pandemic, followed by pharmacies. Common information sources regarding NHPs were the products themselves and pharmacists.

**Conclusion:**

This study emphasized the important role of NHPs as a popular prevention and treatment option for RD. RD-NHPs were more likely used by individuals who were employed, who suffered from a RD and who consult pharmacists for non-fatal health issues. The importance of product information and pharmacies as information sources should be considered to make communication strategies about safe self-medication options with RD-NHPs more effective, which could help to reduce the burden of health facilities regarding non-fatal RD. To improve and develop future pandemic-control strategies, health professionals and policy makers should consider NHP usage behavior and provide critical information about chances and risks of self-medicated NHP consumption.

**Supplementary Information:**

The online version contains supplementary material available at 10.1186/s12906-023-04180-9.

## Background

Respiratory diseases (RD) are widespread and affect all age groups [1]. They are among the most prevalent diseases in primary medical care [2, 3]. On average, each adult experiences 2 or more respiratory infections every year [1]. Common symptoms are for example cough, nasal congestion and secretory symptoms like sneezing and runny nose [2, 4]. Even though the disease progression is often relatively mild to moderate, and ends of its own accord, symptoms can interfere life quality and productivity of sufferers. In 2021, RD were the most common indication on certificates of incapacity to work, and caused one sixth of all cases of incapacity for work in Germany. On average, sick leave due to RD lasted 7.6 days per case of illness [5]. The cost of illness due to RD rose, per German inhabitant, from 190 euros in 2015 up to 230 euros in 2020 [6].

Furthermore, the recent SARS-Covid-19 pandemic demonstrated the potential threat of RD and challenged healthcare systems worldwide [7]. Health facilities became stressed and overloaded. Research and development of new antiviral drugs or alternative medicines got high public attention, aiming to control pandemic consequences like limiting infection rates and implications up to death. However, the development, validation and clinical trials to prove the safety and effectiveness of innovative conventional drugs are laborious and lengthy [8–10]. One complementary approach is the development and use of natural health products (NHPs). They form part of complementary and alternative medicine (CAM) practices. Even if the term NHP is not regulatory defined in Germany, their public perception is comparable to the definition of NHP by the Canadian Government. Thus, NHPs include herbal medicines (HM), homeopathic medicine, vitamins and mineral supplements from a natural source. They can be used, for example, in the treatment, relief or prevention of a disease or its symptoms, or for the maintenance or promotion of health [11]. The regulatory requirements and registration for NHPs differ from those for conventional medicine and vary according to their classification [12, 13]. For example, homeopathic medicine, which is prepared according to the rules of the Homeophatic Pharmacopoeia, is exempt from prescription requirements and over-the-counter (OTC) available [14].

The use of NHPs is traditionally embedded in many cultures and serves as an important part in many healthcare systems [12, 15]. For example, in 2019 more than 75% of the German population took HM over the previous year [16]. In Germany, RD or associated symptoms are the most common indication for NHP usage [14, 16, 17]. Popular herbs, used for RD, include thyme (Thymus vulgaris L.), sage (Salvia officinalis), menthe (Mentha piperita) and garlic (Allium sativum L.) [18–20].

Due to several phytoconstituents, they may have, for example, immune-modulatory or antiviral characteristics, inter alia concerning SARS-CoV-2 or influenza A viruses [19–21].

NHPs mainly constitute healthcare options, which do not require a prescription and are accessible OTC. They have a share of 20% of the total OTC health-product sales [16, 22]. In 2021, pharmacies (including mail order pharmacies) sold around 652 million OTC health-product packages, of which 558 million packages were not prescribed but self-medicated [14]. They were the most common distribution channel for NHPs in Germany [17], followed by drugstores [23]. Before the pandemic, popular sources for information about HM were the internet, pharmacists and family members [16]. Self-medication without the recommendation of health professionals and without consulting them about the self-prescribed NHP intake is a common behavior of many NHP users [24–26]. For example, in 2018, 92% of German HM users took HM in self-medication. Only 38% of these informed their practitioner about the self-medication [16].

Perceived safety of NHPs, e.g. due to their naturalness, can lead to an underestimation of potential side and adverse effects [27, 28]. On the other hand, NHP self-medication can reduce the burden on healthcare systems from financial and capacity perspectives, e.g. by reducing non-mandatory visits to practitioners [29, 30]. Furthermore, self-medication reduces the spending of public health insurance by about 20 million euros per annum in Germany [31]. On an individual level, self-medication can save opportunity-costs, for example arising from practitioner visits, arising from travel time and costs of appointments within normal working hours. Being able to effectively self-care through self-medication can help individuals to prevent delayed or avoided treatment [29].

Given the described high self-medication rate of NHPs, and in order to provide safe healthcare advice, it is necessary to understand consumer NHP use and self-practice [32]. Previous studies suggest that there are more women than men who use NHPs. Furthermore, NHP users were found to be likely middle-aged and well-educated [16, 33, 34]. Depending on the NHP application field, health condition [35] and general health behavior [26] can also be indicators for NHP usage. For example, studies found correlations between vaccination hesitancy or refusal and NHP utilization [36–38]. Moreover, psychological characteristics impact consumer perception and behavior concerning healthcare options [39]. The better a health product meets consumer values and preferences, the more likely it is that the consumer will chose it and the better the adherence to safe usage [40, 41]. Values can feature as criteria and standards, which affect decision-making [42]. According to the Schwartz theory of basic values, there are ten core values, whose relative importance to each other guides attitudes and behavior. They are related and may conflict or correspond to each other. Putting them on two bipolar dimensions, four main value orientations remain. Openness-to-change, including the values of *self-direction, stimulation and hedonism*, is opposed to conservation, which includes the values of *security, conformity and tradition*. On the other scale, self-transcendence, meaning the importance of welfare and interests of others (*universalism and benevolence*) is opposed to the self-focused, self-enhancing values of power and achievement [43].

Altogether, scientific literature already provides some information on NHP usage and its users, especially HM users. However, information has so far been lacking in the specific case of RD. While some studies for Asian and Arabian countries determined NHP usage pattern especially related to the Covid-19 pandemic, little data exist for Western European countries for this period. Although several studies focused on various promising natural compounds concerning their mode of action, efficiency and effectiveness for RD prevention and treatment, or the NHP user in general, little is known about the characteristics of the consumers of RD-NHPs, prevalence of RD-NHP usage or consumer’s usage pattern. Given the high importance of RD for the economy (days absent, costs, etc.), and their potential threat to the healthcare systems as shown by the Covid-19 pandemic, it is important to plan and implement effective strategies and policies for RD epidemic control. Restrictions and regulations in public life lead to changes in health, consumption and information behavior [44, 45]. Scientific knowledge about who uses RD-NHPs and details of their NHP-related behavior could help to develop and optimize future pandemic-control strategies by considering the potential of NHP consumption. Therefore, this study analyses the following research questions: Who uses RD-NHPs in Germany? Which personal and health-behavior-related characteristics explain RD-NHP usage in different user groups? Further, this study aims to disclose NHP-related consumption behavior among RD-NHP users during the Covid-19 pandemic in Germany.

As the study was conducted during the Covid-19-pandemic, the results of this study provide information on NHP usage behavior and the role of RD-NHP use for the healthcare system in a pandemic situation in a Western European country. The insights of this study can help policy makers and healthcare professionals to develop and establish more targeted communication strategies regarding advice on the prevention and safe treatment of RD via self-medicated NHPs in order to reduce the burden on inpatient and outpatient health facilities treating non-fatal RD, as well as to develop and improve coping strategies for future pandemics.

## Methods

### Study design and data collection

We conducted a cross-sectional online survey, representing the general German population (18 years +). A marketing research institute recruited the participants via an online panel and provided monetary compensation of 1 Euro per participant. Because NHPs for RD are used for symptom treatment as well as for disease prevention and health promotion [46–48], we focused on the general German population rather than only on people who suffered from RD within a specific time period. We used quota sampling and set quotas for gender, age, size of place of residence, and federal state to ensure that the distribution of these variables in the sample reflects the distribution of these important sociodemographic characteristics of the German population. Participation requirements were a minimum age of 18 years, a residence in Germany, and sufficient German language skills. Participation was voluntary. The survey contained a standardized questionnaire and was performed in April 2022 in Germany. The 12 months before the survey were entirely within the period of the Covid-19 pandemic. Thus, retrospective questions focusing on the past 12 months pertain entirely to behavior conducted during the Covid-19 pandemic. At the beginning, participants were informed about the purpose of the study, the privacy policy and were able to start the survey after giving their informed consent. A total of 2207 individuals completed the questionnaire. To ensure a reliable and valid dataset, we carefully screened the data according to Schendera 2011 [49]. Participants, who choose always or most times the same response options (“straight-liners”) or who took less time than half of the median time of the whole sample (“speeders”) were excluded. After this initial data cleaning, the sample, which we further analyzed, consisted of 1707 participants. This sample size is sufficient to determine adequate results and prevalence rates with an allowable margin error of < 5% for the objective of the present study [50].

The Ethics Commission of the Faculty of Medicine, Technical University of Munich, approved this study on April 2, 2022.

### Questionnaire design

The questionnaire was based on established items and scales from previous studies, e.g. the short Schwartz value survey (SSVS) by Lindeman and Verkasalo [51], and the results of a qualitative pre-study [52]. It contained two main parts with several sections. The first part dealt with the use of NHPs and was only answered by NHP users. At the beginning of this part, we figured whether an individual had used NHPs. The second part was answered by all participants, and included a section on general health behavior, the SSVS and sociodemographic items. All parts of the questionnaire relevant for this study can be found in Supplementary file 1. To ensure a reliable and valid study instrument, the authors and research assistants critically reviewed the questionnaire. An online pre-test, the results of which are not included in further analyses, was performed to assess general comprehension and timing. Through this procedure, the questionnaire underwent revisions, updates and improvements, until the final version was accepted.

### Questionnaire sections and items

At the beginning of the main part of the questionnaire, participants indicated if they had generally used NHPs, and if ‘yes’, which kind of NHP (prescriptive HM, OTC HM, homeopathic, natural nutritional supplements). Participants with no NHP experience (non-NHP User) skipped the next items and went onto the second part of the questionnaire. Users of NHPs answered further questions about whether they had used NHP within the previous 12 months and about their application fields. Indicating NHP usage within the previous 12 months in the application fields cough, flu/cold or Covid-19 served to define RD-NHP users. Participants who indicated NHP usage in general but not in these application fields were defined as non-RD-NHP users (nRD-NHP users). The subsequent section contained questions about the general aims of NHP usage (health promotion, illness prevention, disease/symptom treatment; multiple answers possible) and NHP-consumption behavior, including self-medication, reporting of self-medication to professionals, and the use of new NHPs since the Covid-19-pandemic (yes/no). Further questions were related to information and supply sources for NHPs within the previous 12 months. All participants answered the second part of the questionnaire. The questions were targeted at general health behavior, for example, on consulting health professionals for non-fatal health issues, whether participants completed preventive medical check-ups, whether they had had a positive Covid-19 test result within the previous 6 months and whether they had an up-to-date influenza, Covid-19 and tetanus vaccination (yes/no). The SSVS-scale contained the 10 core values (Supplementary file 2) described by Schwartz [42], which participants rated on a 7-point-Likert Scale according to their individual importance (against my principles (-1), not important at all (0), up to of supreme importance (5). The last section addressed sociodemographic information like education, occupation status and children living in the same household.

### Statistical analyses

To analyze the sociodemographic characteristics and general health behavior of the RD-NHP users (group 1), nRD-NHP users (group 2) and non-NHP users (group 3) as well as the NHP-related health behavior (e.g. self-medication) of RD-NHP-users, we applied descriptive statistics (frequencies, percentages, mean and SD values). A normal distribution can be assumed due to the sample size. In order to investigate whether there is a significant association between the 3 groups (RD-NHP-user, nRD-NHP-user, non-NHP user) and sociodemographic characteristics or general health behavior, we performed Chi-square tests and calculated Cramer’s V for effect sizes. To analyze value orientations with the SSVS, we followed the suggestions provided by Boer [53]. Therefore, we computed the mean for each value orientation. The value orientation openness-to-change is the mean of the values *hedonism*, *self-direction* and *stimulation*. Conservation maps the mean of the values *conformity*, *tradition* and *security*. Self-enhancement is the mean of *power* and *achievement*. Self-transcendence is the mean of *benevolence* and *universalism*.

As Levene Statistics proved the assumption of homogeneity of variances (p < 0.05) we performed an ANOVA for group comparisons and used ω² for effect size calculations. Following the recommendations by Field, we used Hochberg GT2 for post-hoc comparisons [54]. Additionally, using a multinomial logistic regression, we identified relevant predictors for being a RD-NHP user, an nRD-NHP user or a non-NHP user (dependent variables). Independent variables were sociodemographic and general health behavior related variables, with value orientations entered as covariates. These variables were selected based on insights from scientific literature [16, 53, 55]. Independent variables were checked for outliers and multicollinearity. All Variance Inflation Factors were < 2, thus there was no collinearity problem in the data [54]. All significance tests were performed on a p < 0.05 level. Calculations were performed using the software IBM Statistics SPSS for Windows, release 29.

## Results

Of the 1707 participants, 14.2% answered that they had never used NHPs (non-NHP user), 1464 participants indicated experience with NHPs. 794 participants (46.5%) had used RD-NHPs within the past 12 months (RD-NHP user); 670 participants (39.3%) had generally used NHPs, but no RD-NHPs in the past 12 months, which we defined as the nRD-NHP user group. Among the general NHP users (n = 1464), 54.2% declared NHP usage for RD in the past 12 months, 45.8% had not used NHPs for RD in this timeframe.

### Sociodemographic characteristics and general health behavior

Table 1 provides the distribution of sociodemographic characteristics of the total sample and of the three groups.

**Table 1:** Sociodemographic characteristics of study participants and Chi-square test results; n = 1707

Variables	Total sample	RD-NHP user,	nRD-NHP user,	Non-NHP-user	X²(df); p-value	Cramer’s V
Total	100% (n = 1707)	46.5% (n = 794)	39.3% (n = 670)	14.2% (n = 243)		
*Gender*						
Male	46.9% (n = 801)	39.9% (n = 317)	47.0% (n = 315)	69.5% (n = 169)	X²(4) = 65.56, p = < 0.001	
Female	53.1% (n = 906)	60.1% (n = 477)	53.0% (n = 355)	30.5% (n = 77)		0.196
*Age (years)*						
18–29	14.6% (n = 250)	17.4% (n = 138)	13.6% (n = 91)	8.6% (n = 21)	X²(8) = 52.95, p = < 0.001	
30–59	48.6% (n = 829)	52.5% (n = 417)	48.4% (n = 324)	36.2% (n = 88)		0.125
60 +	36.8% (n = 628)	30.1% (n = 239)	38.1% (n = 255)	55.1% (n = 134)		
*Education (years)*						
< 12 years	49.6% (n = 847)	44.1% (n = 350)	52.2% (n = 350)	60.5% (n = 147)	X²(4) = 65.56, p = < 0.001	
>= 12 years	50.4% (n = 860)	55.9% (n = 444)	47.8% (n = 320)	39.5% (n = 96)		0.116
*Occupation*						
Unemployed	37.1% (n = 633)	29.7% (n = 236)	40.0% (n = 268)	53.1% (n = 129)	X²(4) = 47.55, p = < 0.001	
Employed	62.9% (n = 1074)	70.3% (n = 558)	60.0% (n = 402)	46.9% (n = 114)		0.167
*Children in household*						
Yes	19.4% (n = 331)	25.7% (n = 204)	14.9% (n = 100)	11.1% (n = 27)	X²(4) = 39.38, p = < 0.001	
No	80.6% (n = 1376)	74.3% (n = 590)	85.1% (n = 570)	88.9% (n = 216)		0.152
*Federal State*						
East	15.9% (n = 271)	16.9% (n = 134)	12.5% (n = 84)	21.8% (n = 53)	X²(2) = 12.60, p = 0.002	
West	84.1% (n = 1436)	83.1% (n = 660)	87.5% (n = 586)	78.2% (n = 190)		0.086

More women than men used NHPs and RD-NHPs in the previous 12 months. The share of individuals who are middle-aged, well-educated, employed and having children living in the same household was highest in the RD-NHP user group, followed by the nRD-NHP user group and lowest in the group of non-NHP users. The non-NHP user group differed from both NHP-using groups with a significant lower share of middle-aged individuals. With more than half of the individuals having 12 or more years of school education, the proportions of school-education were significantly different for RD-NHP users compared to the other groups, which had higher shares for less than 12 years of education. Chi-square tests proved a significant difference of all tested sociodemographic variables between NHP user groups (p < 0.05). According to Cohen, effect sizes were small (Cramer’s V < 0.03) [56].

**Table 2:** Health related behavioral characteristics of NHP user groups; n = 1707

Variables	Total sample	RD-NHP user	nRD-NHP user	Non-NHP user	X²(df); p-value	Cramer’s V
Total (%)	100% (n = 1707)	46.5% (n = 794)	39.3% (n = 670)	14.2% (n = 243)		
*Covid-19 infection*						
Yes	16.4% (n = 280)	23.0% (n = 183)	9.6% (n = 64)	13.6% (n = 33)	X²(2) = 0.49.91, p = < 0.001	
No	83.6% (n = 1427)	77.0% (n = 611)	90.4% (n = 606)	86.4% (n = 210)		0.171
*Influenza vaccination*						
Yes	34.8% (n = 594)	32.7% (n = 260)	35.5% (n = 238)	39.5% (n = 96)	X²(2) = 1.25, p = 0.264	
No	65.2% (n = 1113)	67.3% (n = 534)	64.5% (n = 432)	60.5% (n = 147)		0.048
*Covid-19 vaccination*						
Yes	85.4% (n = 1458)	85.8% (n = 681)	85.4% (n = 572)	84.4% (n = 205)	X²(2) = 0.30, p = 0.862	
No	14.6% (n = 249)	14.2% (n = 113)	14.6% (n = 98)	15.6% (n = 38)		0.013
*Tetanus vaccination*						
Yes	66.7% (n = 1138)	69.8% (n = 554)	64.6% (n = 433)	62.1% (n = 151)	X²(2) = 6.94, p = 0.031	
No	33.3% (n = 569)	30.2% (n = 240)	35.4% (n = 237)	37.9% (n = 92)		0.064
*Preventive check-ups*						
Yes	65.7% (n = 1121)	69.0% (n = 548)	64.6% (n = 433)	57.6% (n = 140)	X²(2) = 11.27, p = 0.004	
No	34.3% (n = 586)	31.0% (n = 246)	35.4% (n = 237)	42.4% (n = 103)		0.081
*Health consultants*						
General practitioner	80.5% (n = 1374)	82.2% (n = 653)	75.7% (n = 507)	88.1% (n = 214)	X²(2) = 20.34, p = < 0.001	0.109
Pharmacist	23.1% (n = 395)	29.7% (n = 236)	20.7% (n = 139)	8.2% (n = 20)	X²(2) = 51.88, p = < 0.001	0.174
Alternative practitioner	6.6% (n = 113)	9.3% (n = 74)	5.7% (n = 38)	0.4% (n = 1)	X²(2) = 25.49, p = < 0.001	0.122
Family/friends	26.1% (n = 445)	31.6% (n = 251)	24.8% (n = 166)	11.5% (n = 28)	X²(2) = 39.92, p = < 0.001	0.153
Nobody	5.6% (n = 96)	4.3% (n = 34)	7.5% (n = 50)	4.9% (n = 12)	X²(2) = 7.18, p = 0.028	0.065

Table 2 shows health behavior related characteristics, including vaccination statuses and health consultants for non-fatal health issues of the different NHP groups. Chi-square tests gave evidence of a significant difference between NHP user groups and Covid-19 infection, tetanus vaccination, preventive check-ups, and all types of health consultants, but not for influenza and Covid-19 vaccination. Effect sizes were small (Cramer’s V < 0.03) [56].The share of individuals who had had a Covid-19 infection within the previous six months was higher among RD-NHP users (23.0%) than in the nRD-NHP user group (9.6%). Tetanus vaccination was more often up-to-date in the RD-NHP user group compared to the other groups. More RD-NHP and nRD-NHP users attended preventive medical check-ups than non-NHP users, and a higher percentage of them consulted pharmacists, alternative practitioners and family or friends for health advice in case of non-fatal health issues. The share of individuals who consulted family and friends was significantly higher among both NHP-using groups compared to the non-NHP using group. In contrast, a higher percentage of non-NHP users consulted their general practitioner for health advice than NHP users did. Here, the share of nRD-NHP users was significantly lower than among the other groups, but with 75.7% still high compared to the other consultant options.

The results for the value orientation expression of the three NHP user groups, including the orientation characteristic regarding openness-to-change, conservation, self-enhancement and self-transcendence, are shown in Table 3.

**Table 3:** Value orientations of NHP user groups; n = 1707

		User group means (SD)					
Variables	Total sample	RD-NHP user (1)	nRD-NHP user (2)	Non-NHP user (3)	ANOVA (F-Ratio)	Hochberg GT2 post-hoc	ω²
Total (n)	1707	794	670	243			
Openness-to-change	2.86 (1.01)	2.99 (0.99)	2.84 (0.99)	2.47 (1.04)	26.17***	1|2; 1|3; 2|3	0.03
Conservation	3.16 (1.01)	3.15 (0.98)	3.19 (1.02)	3.12 (1.05)	0.43		
Self-transcendence	3.90 (0.98)	3.95 (0.99)	3.91 (0.97)	3.69 (0.98)	7.05***	1|2; 1|3	0.01
Self-enhancement	1.44 (1.26)	1.57 (1.30)	1.41 (1.21)	1.14 (1.24)	11.30***	1|2; 1|3	0.01

Scale: -1 against my principles, 0 not important at all, 1 not important, 2 rather not important, 3 rather important, 4 important, 5 of supreme importance; ***p ≤ 0.001; post-hoc significant differences between: 1 = RD-NHP user group, 2 = nRD-NHP user group; 3 = Non-NHP user group, p ≤ 0.05

Most important for all groups was self-transcendence, and the least important was self-enhancement. In between, conservation was more important than openness-to-change. The ANOVA test indicates significant differences between the groups concerning openness-to-change, self-enhancement and self-transcendence. The omega square values close to 0 indicate negligible effect sizes. The result of the post hoc tests shows significant differences between all groups for the openness-to-change value orientation, according to the definition of value orientations by Schwartz [43]. Non-NHP users were less open than NHP users, and RD-NHP users were more open than nRD-NHP users. For self-transcendence and self-enhancement, the post hoc tests indicate significant differences between non-NHP users and both NHP user groups (RD-NHP user, nRD-NHP user), but no differences between RD-NHP user and nRD-NHP user. Self-enhancement and self-transcendence were both less important for non-NHP users than for the NHP-user groups.

### Variables influencing differences in RD-NHP usage

Using multinomial logistic regression, we analyzed relevant predictors for being an RD-NHP user, an nRD-NHP user or a non-NHP user. Results are shown in Table 4. The likelihood ratio test was statistically significant at p < 0.001 (χ² (42) = 337.228). The Nagelkerke’s R² of 0.207 indicates an acceptable amount of explained variance [57].

**Table 4:** Results of the multinomial logistic regression to identify predictors of RD-NHP consumption in the previous 12 months; n = 1707

	nRD-NHP user			Non-NHP-user		
Variables	B	OR	95% CI	B	OR	95% CI
Gender (male)	0.21	1.23	0.98–1.55	1.08***	2.93	2.08–4.13
Age 18–29 (young)	0.19	1.21	0.82–1.79	0.15	1.16	0.63–2.14
30–59 (middle-aged)	0.12	1.13	0.85–1.50	-0.35	0.70	0.47–1.06
Education < 12 years	0.19	1.21	0.98–1.55	0.41*	1.51	1.09–2.10
Occupation unemployed	0.30*	1.35	1.04–1.75	0.54**	1.71	1.18–2.47
Children in household (no)	0.45**	1.57	1.17–2.11	0.24	1.27	0.78–2.09
Federal State (West)	0.44**	1.55	1.14–2.11	-0.24	0.79	0.53–1.17
Covid-19 infection (no)	0.96***	2.61	1.90–3.60	0.33	1.40	0.89–2.19
Influenza vaccination (no)	-0.05	0.96	0.74–1.23	0.04	1.04	0.72–1.50
Covid-19 vaccination (no)	0.13	1.14	0.83–1.58	0.24	1.27	0.80–2.02
Tetanus vaccination (no)	0.15	1.16	0.90–1.48	0.23	1.26	0.90–1.79
Preventive check-ups (no)	0.02	1.02	0.79–1.31	0.39	1.47	1.04–2.09
*Health consultants*						
General practitioner (yes)	-0.39*	0.68	0.50–0.93	0.60*	1.82	1.03–3.22
Pharmacist (yes)	-0.34**	0.71	0.55–0.92	-1.20***	0.30	0.18–0.50
Alternative practitioner (yes)	-0.43	0.65	0.42-1.00	-2.55*	0.08	0.01–0.58
Family and Friends (yes)	-0.18	0.84	0.65–1.08	-0.81***	0.44	0.28–0.71
Nobody (yes)	0.13	1.14	0.66–1.97	0.31	1.36	0.55–3.34
*Value orientations*						
Openness to change	-0.09	0.91	0-80-1.04	-0.28**	0.75	0.63–0.90
Conservation	0.10	1.10	0.98–1.24	0–10	1.11	0.93–1.31
Self-enhancement	-0.01	0.99	0.90–1.09	-0.09	0.92	0.80–1.06
Self-transcendence	-0.02	0.98	0.86–1.12	-0.06	0.95	0.79–1.13

Reference group: RD-NHP user; reference categories: gender female; age 60+; children in household yes; federal state East; Covid-19 infection, influenza vaccination, Covid-19 vaccination, tetanus vaccination, preventive check-ups: yes; Health consultants: no. * p ≤ 0.05; ** p ≤ 0.01; *** p ≤ 0.001; bold data are significant

Gender, age and education did not significantly influence the likelihood of being an nRD-NHP user compared to being a RD-NHP user. Being unemployed, living without children in the same household and a residence in a western federal state significantly increased the likelihood of being an nRD-NHP user rather than a RD-NHP user. Furthermore, having suffered from a Covid-19 infection in the past 6 months had a significant effect on the likelihood of being an nRD-NHP-user compared to RD-NHP users.

Consulting the general practitioner for non-fatal health issues was a behavior significantly less likely for nRD-NHP user but significantly more likely for non-NHP users than for RD-NHP users. The likelihood of consulting pharmacists was significantly lower for both non-RD-NHP using groups compared to the RD-NHP user group. Consulting alternative practitioners, family and friends were less likely for non-NHP users than for RD-NHP users. Men were more likely to be non-NHP users than RD-NHP users. Compared to RD-NHP users, people who had never taken NHP were additionally more likely to be unemployed and to have less than 12 years of school education. Openness-to-change was the only value orientation found to be a predictor for the likelihood of being a non-NHP user compared to RD-NHP users. Stronger value orientation towards openness-to-change values increased the likelihood of being a RD-NHP user compared to not using NHPs at all.

The likelihood of being an nRD-NHP or a non-NHP user compared to a RD-NHP user was not significantly influenced by general health behavior, including up-to-date vaccination statuses.

### RD-NHP consumption behavior during the Covid-19 pandemic

To examine the NHP consumption behavior of RD-NHP users (n = 794) in Germany during the Covid-19 pandemic in more detail, we analyzed questions regarding their NHP consumption behavior since the beginning of the pandemic. Table 5 shows general NHP usage aims, frequencies of NHP-related health behavior, and self-medication practices.

**Table 5:** Frequency table regarding NHP-related behavior of NHP-consumption of RD-NHP users during the Covid-19 pandemic (n = 794)

Variables		RD-NHP user		
Total		100% (n = 794)		
*Aims¹*				
Health support/ maintenance		79.1% (n = 628)		
Disease prevention		57.9% (n = 460)		
Diseases/symptom treatment		77.6% (n = 616)		
*New NHP since Covid-19*				
Yes		18.4% (n = 146)		
No		81.6% (n = 648)		
*Change in NHP usage amount*				
More NHPs		13.2% (n = 105)		
No change		82.4% (n = 654)		
Fewer NHPs		4.4% (n = 35)		
*NHP self-medication*				
Yes		89.4% (n = 710)		
No		10.6% (n = 84)		
*Informed practitioner about NHP self-medication²*				
Yes		32.1% (n = 228)		
No		67.9% (n = 482)		

¹ multiple answers allowed ; ²Total n = 710

Most RD-NHP users took NHPs to support or maintain (79.1%) their health, followed by the aim of treating diseases or symptoms (77.6). 57.9% mentioned disease prevention as one aim of NHP usage. 18.4% of RD-NHP users tried new NHPs which they had not used before the Covid-19 pandemic. Most RD-NHP users noticed no change in their general NHP usage frequency (82.4%). Nevertheless, compared to the 4.4% of RD-NHP users who took fewer NHPs since the time before the pandemic, 3 times more RD-NHP users (13.2%) took more NHPs than before the Covid-19 pandemic.

Most RD-NHP users consumed NHPs in self-medication (89.4%). 67.9% of them did not inform their practitioner about the self-medication.

**Fig. 1:**
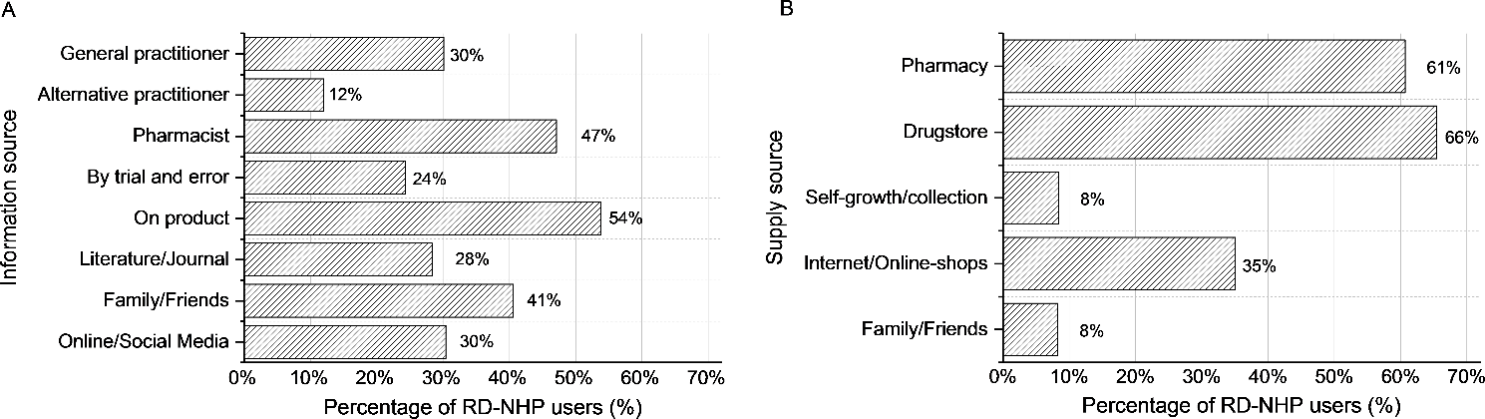
Frequencies of (**A**) information sources about NHPs and (**B**) NHP supply sources in the previous 12 months; n = 794; multiple answers allowed

Figure 1 shows which sources RD-NHP users used to obtain information about NHPs in the past 12 months, as well as where they purchased NHPs within the past 12 months. More than half of RD-NHP users (53.8%) obtained information about an NHP from the product itself, e.g. by studying the packaging and reading the declaration. Pharmacists were mentioned by 47.1% of RD-NHP users as an information source for NHPs, followed by family and friends (40.6%) and the internet or social media (30.4%).

At 58.8%, drugstores were the most often mentioned supply source for NHPs in the past 12 months, followed by pharmacies (54.4%) and the internet/online-shops (35.3%). Self-growth or -collection of NHPs (8.4%) or getting them from family and friends (8.3%) was less common among RD-NHP users.

## Discussion

RD are widespread and can, as recently shown in the Covid-19 pandemic, burden healthcare facilities worldwide. In this study, we examined the role of NHPs for RD in the German population by conducting a cross-sectional representative online survey. Our study gives insights into which sociodemographic and health behavior-related characteristics predict RD-NHP usage and discloses the NHP consumption behavior of RD-NHP users during the Covid-19 pandemic.

The results show a lifetime prevalence rate for general NHP usage of 85.8%, which is analogous to previous findings for HM usage prevalence (86.7%) found in 2018 in Germany [16]. The 12-months prevalence rate for RD-NHP usage was 46.5%. Comparable prevalence rates have been found e.g. in the Iranian [58] and Vietnamese population [59].

The logistic regression analyses indicated gender (female) and higher education as predictors for RD-NHP usage in relation to non-NHP usage, which is in line with previous findings [35, 60, 61]. According to the results of our study, age was no predictor for RD-NHP usage. However, employment was a predictor for RD-NHP usage compared to nRD-NHP and non-NHP usage. The tendency to go to work despite being ill could be linked to influenza-related behavior [62]. Supporting health or treating RD symptoms to stay fit enough for work could encourage the use of NHPs, especially when they are sold OTC, and as such easily accessible and available without practitioner consultation [29]. While self-medicated RD-NHP usage might help users to save money, e.g. reducing the financial costs due to less missing work [29], it may lead to underestimation of the disease and facilitate presentism. Presentism, in the form of working despite being sick, can lead to less productivity by the individual, worsen the disease or symptoms and result in longer sick leaves. Further, the risk of infection for other employees might raise [5]. However, in Germany employees are entitled to get 6 weeks of employer-paid sick leave, which lead to no financial wage loss of employed people due to the absence of work caused by illness within this timeframe [63].

Suffering a Covid-19 infection in the previous 6 months was a strong predictor of RD-NHP usage compared to nRD-NHP usage. This finding implies RD-NHP usage for the treatment of Covid-19 symptoms during the pandemic, which is in line with findings from research about the concrete connection of NHP/HM use to the indication of Covid-19 [8, 9, 48, 64, 65].

Our study found no relationship between vaccination behavior and NHP usage. This finding is in contrast to previous research, which indicated links between NHP usage and vaccination endorsement [66]. For example, receiving a flu vaccination [67] or a Covid-19 vaccination [68, 69] was less likely for people who favor NHP over conventional drugs. It might be possible that the kind of NHP a person consumes makes a difference to vaccination behavior. One study among Australian women found that the consultation of alternative practitioners and consuming herbal medicines reduced the probability of influenza vaccinations, while consuming nutritional supplements increased the likelihood of influenza vaccination [70].

Regarding the value orientation, we found stronger openness-to-change values to be more likely for RD-NHP users than for non-NHP users. This is in line with previous studies, which found that the personality characteristic of openness was linked to the use of CAM [39, 71, 72]. The relation of the strength of value orientations we found is consistent to previous findings concerning the general German population, which also determined self-transcendence as the most important value orientation and self-enhancement as the least important, with conservation stronger than openness-to-change in between the former two [55].

Most NHP users in our study indicated no change regarding their NHP consumption during the pandemic. Nevertheless, the share of RD-NHP users who consumed more NHPs than before the pandemic was, at 13.2%, higher than the share of users who took fewer NHPs (4.4%). For other countries, studies show different results. For example, studies in Saudi Arabia indicated increased NHP consumption during the pandemic [65, 73]. In contrast, for Hong Kong a decrease in NHP consumption was found [74]. On the one hand, fewer non-Covid-19 respiratory infections, e.g. caused by influenza viruses, could have led to a decreased RD-NHP demand [75]. On the other hand, the urge to support and maintain health, which we found was one NHP-consumption reason of 79.1% of RD-NHP users, could be an explanation for increased RD-NHP consumption during the pandemic.

Our study showed a high self-medication rate of 89.4% among RD-NHP users during the pandemic. This rate is comparable to the 93% self-medication prevalence found for general HM users before the pandemic in 2018 [16]. It confirms the prediction of no significant change in self-medication during the Corona pandemic, as assumed in literature reviews [76, 77].

Further, the results of our study showed that 32.1% of respondents who used RD-NHP informed their medical practitioner about the consumption of self-prescribed NHPs. This rate is lower than the rate found for HM usage in Germany in 2018 [16]. During the Covid-19 pandemic, patients avoided practitioner consultation for non-urgent health issues [78], and visits to the general practitioner decreased [79–81]. A positive aspect of self-medication with NHPs is the relief of primary healthcare facilities, e.g. general practitioners, who could focus more on patients with urgent and serious health issues. However, one problem which has been frequently discussed in literature is the lack of risk-awareness concerning NHP consumption. Users might deem NHP to be safe, e.g. because of their naturalness, and underestimate their side-effects or drug-interactions [16, 67, 82, 83]. However, appropriate and responsible self-medication with medically-approved and -recommended NHPs can provide benefits for their users and the healthcare system as a whole [29].

Drugstores were the most often mentioned supply source of RD-NHP users for NHPs in our study. In general, drugstores do not provide personal healthcare advice for the NHPs sold OTC [84]. Our study showed that 53.8% of RD-NHP users checked the product packaging for information about their NHP. This emphasizes the importance of a clear and transparent declaration. For the effective communication of critical information, such as recommended NHP adherence and potential risks, it is necessary to provide information where users request it.

Mentioned by 47.1% of RD-NHP users as an information source and by 60.7% as the second most frequent supply source, our study underlines the important role of pharmacists as a consultation option for non-fatal health issues. Professional health advice from pharmacists can help NHP users to make informed decisions [85, 86]. Pharmacists were more likely to be consulted by RD-NHP users than by nRD-NHP users or non-NHP users. Their importance in helping to control the Covid-19 pandemic was found in further studies, conducted around the globe [32, 87–89]. In our study, 12% of RD-NHP users asked alternative practitioners for information about NHPs. This is much lower than in Vietnam, for example, where more than 40% consulted alternative practitioners [59]. Such a variance in consulting prevalence could be due to differences in healthcare systems between European and Asian cultures [90]. Irrespective of the consultation prevalence, all healthcare professionals from pharmacists to general and alternative practitioners should have easy access to evidence-based, up-to-date information about NHPs to be able to advise their clients regarding save NHP usage. For further evaluation of safety and efficacy of NHP, health professionals should be provided with knowledge and resources to gather information about the NHP consumption experience of their patients and to report any medical-related problems like adverse effects to manufacturers and regulatory authorities. Further issues about this aspect are discussed by Sharma et al. 2017 [91, 92].

Interestingly, our findings indicate relatively few NHP users searching the internet or social media for information about NHPs (30%). This rate is similar to the results of studies conducted in Vietnam [59] and Saudi Arabia [73] during the Covid-19 pandemic, but represents fewer than half of the 68.2%, which a study in Germany revealed as using the internet as an information source for HM in 2018 [16]. Another important non-professional source for information about NHPs considered by RD-NHP users were friends and family members (41%). This result is comparable to the findings for HM information sources in Germany before the pandemic [16]. Consequently, we can assume that family and friends got neither more nor less requests for NHP information during the Covid-19 pandemic. In sourcing NHPs, our findings indicated a minor role for family and friends as well as for self-growing or collecting practices. This is different to the results of a study in Saudi Arabia, which found that NHPs from home were the primary product source, followed by drugstores and pharmacies [93]. Those differences could be caused inter alia by culture and the local healthcare system, which also impact NHP consumption behavior [28, 90, 94].

Finally, we address the strengths and limitations of this study. This study provides a valuable contribution to a better understanding of general RD-NHP usage within the Covid-19 pandemic. It is one of the first studies, which analyzed the interrelationship between RD-NHP and NHP usage within a pandemic context. However, this study did not determine the specific respiratory symptoms or diseases to distinguish RD-NHP user accordingly, for example if RD-NHP user took RD-NHP for an influenza or tuberculosis infection.

It is to mention, that sampling via an online-panel carries the risk of certain bias [95]. It cannot guarantee full representativeness, as people without internet access could not join the study. Even if interest or usage of NHP was not obligatory to participate in this study, the sample could be biased towards participants with NHP experience or interest. Thus, selection bias could occur and lead to biased and overestimated prevalence rates. Nevertheless, one strength of the present study is that the sample had a sufficient size to calculate prevalence rates with small errors [50]. To address the issue of representativeness, the study contained pre-quotation for four sociodemographic variables to picture their distribution of the German population. Furthermore, even though we carefully screened the data before analysis to ensure data quality, we cannot completely eliminate errors or recall bias in participants’ answers. To classify participants, we analyzed a retrospective question regarding whether or not participants had consumed NHPs for RD within the previous 12 months. The prefixed question contained the product types (e.g. HM, natural nutrition supplements, etc.) to disclose the definition of NHPs. Nevertheless, we cannot rule out that some participants may have had products in mind, which were outside the scope of our definition of NHPs. Accordingly, incorrect classifications in the user groups cannot be excluded. Further, the definition of NHPs and their components, such as HM, is not consistent in literature. The definition of the included items into the definition of NHP or HM might slightly differ. Overall consistency applies for the condition of naturalness for included products. Using one regulatory definition of NHP, as we did in our study, provides better chances of comparability with further studies, which refer to the same definition. Due to different healthcare systems and payment requirements for NHPs, the results of our study might not be transferable to other countries and cultures. NHP usage pattern can be related to cultural and traditional embedment of CAM, costs and access availabilities as well as the resources of and for conventional health care options [16, 39, 71, 96, 97].

## Conclusion

The recent pandemic showed the vulnerability of healthcare systems to RD in terms of being challenged by new infectious RD like the SARS-Covid-19 virus. Our study emphasized the important role of NHPs as a popular prevention and treatment option for RD in Western European countries like Germany. The results of this study showed, that employed people who suffered from a RD and who often consult pharmacists for non-fatal health issues, were likely to use RD-NHPs. Vaccination-related behavior did not predict RD-NHP usage, which might encourage future research to reassess assumptions regarding NHP consumption and vaccination behavior. Most users of RD-NHP consumed NHP in self-medication, which might have helped to relieve the burden on healthcare facilities at least somewhat. Nevertheless, only a small proportion of participants informed their health professional about their self-medication with NHPs.

Drugstores were a very popular supply source, and users often obtained information by reading the information on and in the package itself. Healthcare professionals should actively ask about self-medicated NHPs, as NHPs can have side-effects and interaction effects. To evaluate individual chances and the risks of NHP usage, health professionals need training, knowledge and easy accessible up-to-date information about NHPs.

Prospective research is needed to examine personal and health-behavior-related characteristics of users who take NHP in order to respiratory symptoms related to specific diseases. Further, future research can take the results of our study into account to determine correlations to the number of deaths caused by Covid-19. Moreover, future studies with a similar research design to our study, conducted in different countries, could provide further international comparability of our results. The insights into NHP consumption behavior in a pandemic situation in a Western European country can help policymakers and healthcare professionals to develop and optimize future pandemic-control strategies, by taking health and information behavior of the population into account. For example, future pandemic control strategies could consider the finding of the importance of on product information and pharmacies as information sources. Effective communication strategies about save self-medication options for RD-NHPs and their recommended safe application by the consumers could help to reduce the burden of inpatient and outpatient health facilities with regard to non-fatal RD. To ensure and evaluate the efficacy of new and established NHPs, NHP consumers and healthcare professionals should be enabled to report NHP usage experiences to health- and pandemic regulatory authorities. Altogether companies in the field of NHP profit from the insights in different groups of consumers and can fine-tune their business strategies accordingly not least in case of unexpected events like e.g. a pandemic.

### Electronic supplementary material

Below is the link to the electronic supplementary material.

Supplementary Material 1

Supplementary Material 2

## Data Availability

The datasets used and/or analyzed during the current study are available from the corresponding author on reasonable request.

## Abbreviations

NHP: Natural health product
RD: Respiratory disease
HM: Herbal medicine
nRD-NHP: NHP not for RD indications
CAM: Complementary and alternative medicine
OTC: over-the-counter
SSVS: Short Schwartz value survey
SD: Standard deviation
