# Supplementary material for: Usage of Natural Health Products (NHPs) for respiratory diseases: user characteristics and NHP-Consumption behavior during the Covid-19 pandemic in Germany
Source: BMC Complement Med Ther. 2023 Oct 21;23:372. doi: 10.1186/s12906-023-04180-9 (PMC10589963; doi:10.1186/s12906-023-04180-9)
Supplement: Supplementary file 1 — Supplementary Material 1 [file 12906_2023_4180_MOESM1_ESM.docx]

**Supplementary file 1: Excerpt from the questionnaire**

Questions relevant for the Study *“Usage of Natural Health Products (NHPs) for Respiratory Diseases: User Characteristics and NHP-Consumption Behavior during the Covid-19 Pandemic in Germany”*

**Quotation-Questions**

How old are you

- 18-29
- 30-39
- 40-49
- 50-59
- 60 years +

What is your gender?

- male
- female
- divers

In which federal state is your residence?

- o Bavaria
- o Baden-Wuerttemberg
- o Berlin
- o Brandenburg
- o Bremen
- o Hamburg
- o Hesse
- o Mecklenburg-Western Pomerania
- o Lower Saxony
- o North Rhine-Westphalia
- o Rhineland-Palatinate
- o Saarland
- o Saxony
- o Saxony-Anhalt
- o Schleswig-Holstein
- o Thuringia

How many people live in your residence town?

- less than 5.000
- 5.000 to <20.000
- 20.000 to <100.000
- 100.000 to <500.000
- 500.00 or more

**PART 1: NHP consumption**

Which natural health products have you ever used? (multiple answers allowed)

- prescriptive herbal medicine
- over-the-counter herbal medicine
- foods declared as supporting health (e.g. juices)
- natural nutritional supplements
- herbal homeopathic remedies
- other: _______________
- I have never used natural health products. *(exclusive answer/no other answer possible 🡪 skip to part 2)*

Have you used natural health products during the past 12 months for your own health or illness?

Natural health products include all products that are made from natural ingredients and aim to have a positive impact on health and well-being. These include herbal medicines, natural nutrition supplements and preparations from plants such as health teas.

- yes
- no

For which of the following health problems have you used natural health products in the last 12 months to treat diseases/symptoms or support your health? (multiple answers allowed)

- Common cold/flu infection
- Covid-19
- Cough
- Insomnia
- Anxiety/restlessness
- Depression
- Concentration/Cognition
- Dementia
- Headaches/migraines
- Pain
- Tinnitus
- Blood pressure
- other: ____________

Which of the following is/are your goal/s for the consumption of natural health products? (multiple answers allowed)

- Health support/ maintenance
- Disease prevention
- Diseases/symptom treatment

Have you also used natural health products in self-medication in the last 12 months without prescription or recommendation by a physician?

- yes
- no

Have you informed your general practitioner or health care provider about your use of natural health products in self-medication?

- yes
- no

How did your consumption of natural health products change since the beginning of the Covid-19 pandemic in Germany?

I take…

- more
- the same amount of
- fewer

…natural health products than before the pandemic.

Do you use natural health products that you did not use before the Corona pandemic?

- yes
- no

Where have you obtained natural health products within the previous 12 months? (multiple answers allowed)

- Pharmacy
- Drugstore (e.g. dm, Rossmann, Müller)
- Selfgrowth or self-collection
- Internet/Onlineshops
- Familiy/Friends
- other: _____________

Where do you inform yourself about the effectiveness and possible areas of application of natural health products? (multiple answers allowed)

- General practitioner
- Alternative practitioner
- Pharmacist
- By trial and error
- On product
- Literature/journal
- Family/Friends
- Online/Social Media
- other: __________

===========================

**PART 2:**

**Health Behavior**

In the following section, you will now be asked questions about your general health and health behavior.

Please answer all questions conscientiously and honestly. There are no right or wrong answers. All data in this survey is collected anonymously, which means that no conclusions can be drawn about you personally.

Who do you consult if you have (non-life-threatening) health complaints? (multiple answers allowed)

- General practitioner
- Pharmacist
- Alternative practitioner
- Family
- Friends
- Nobody
- other: _______________

Please select whether or not the following statements apply to you:

- Within the last 12 months, I have received a flu vaccination.
- Within the last 6 months, I have received a Covid-19 vaccination.
- My tetanus vaccination is up to date.
- I join the recommended preventive medicinal check-up (e.g. cancer screening, birthmark control …).

Have you had a positive Covid-19 test result within the previous 6 months?

- yes
- no

**Short Schwartz Value Survey (SSVS)**

Please read through the following values and their descriptions.

Then tick how important these values are for you personally.

| Value | Against my values | Not important at all | Not important | Rather not important | Rather important | important | Of supreme importance |
| --- | --- | --- | --- | --- | --- | --- | --- |
| **Power**: social status and prestige, control or dominance over people and resources |  |  |  |  |  |  |  |
| **Achievement:** Personal success through demonstrating competence according to social standards |  |  |  |  |  |  |  |
| **Hedonism:** Pleasure and sensuous gratification for oneself |  |  |  |  |  |  |  |
| **Stimulation:** Excitement, novelty, and challenge in life |  |  |  |  |  |  |  |
| **Self-Direction:** Independent thought and action, choosing, creating, and exploring |  |  |  |  |  |  |  |
| **Universalism:** Understanding, appreciation, tolerance, and protection for the welfare of *all* people and of nature |  |  |  |  |  |  |  |
| **Benevolence:** Preservation and enhancement of the welfare of people with whom one is in frequent personal contact |  |  |  |  |  |  |  |
| **Tradition:** Respect, commitment, and acceptance of the customs and ideas that traditional culture or religion provides |  |  |  |  |  |  |  |
| **Conformity:** The restraint of actions, inclinations, and impulses that are likely to upset or harm others and violate social expectations or norms |  |  |  |  |  |  |  |
| **Security:** Safety, harmony, and stability of society, relationships, and self |  |  |  |  |  |  |  |

**Sociodemographic data**

Wat is your highest educational achievement?

- Without graduation
- Secondary modern school or equivalent
- Graduation from polytechnical school in GDR
- School leaving graduation
- Bachelor
- Diploma
- Master
- Ph.D.
- Alternative degree:______________

How is your occupation status?

I am…

- Full-time employed
- Part-time employed
- Partial retirement
- Marginally employed
- Not employed (including pupils or students, job seekers or unemployed, early retirees, pensioners)
- In vocational training
- Retirement
- Federal voluntary service/voluntary social year
- Maternity, parental leave, parental leave

How many people live permanently in your household, including yourself?

This includes all persons with whom you live and manage together.

- One person
- More than one person

Counting me in, ___ people are living in my household, including ___ children aged under 18 years.
