# Supplementary material for: Usage of Natural Health Products (NHPs) for respiratory diseases: user characteristics and NHP-Consumption behavior during the Covid-19 pandemic in Germany
Source: BMC Complement Med Ther. 2023 Oct 21;23:372. doi: 10.1186/s12906-023-04180-9 (PMC10589963; doi:10.1186/s12906-023-04180-9)
Supplement: Supplementary file 2 — Supplementary Material 2 [file 12906_2023_4180_MOESM2_ESM.docx]

**Supplementary file 2**

10 core values and their description by Schwartz (43)

| Value | Description |
| --- | --- |
| Power | Social status and prestige, control or dominance over people and resources. |
| Achievement | Personal success through demonstrating competence according to social standards. |
| Hedonism | Pleasure or sensuous gratification for oneself. |
| Stimulation | Excitement, novelty and challenge in life. |
| Self-direction | Independent thought and action—choosing, creating, exploring. |
| Universalism | Understanding, appreciation, tolerance, and protection for the welfare of all people and for nature. |
| Benevolence | Preserving and enhancing the welfare of those with whom one is in frequent personal contact (the ‘in-group’). |
| Tradition | Respect, commitment, and acceptance of the customs and ideas that one's culture or religion provides. |
| Conformity | Restraint of actions, inclinations, and impulses likely to upset or harm others and violate social expectations or norms. |
| Security | Safety, harmony, and stability of society, of relationships, and of self. |

43. Schwartz SH. An overview of the Schwartz theory of basic values. Online readings in Psychology and Culture. 2012;2(1):2307-0919.1116
